# VenomPred 2.0: A Novel *In Silico* Platform for an Extended and Human Interpretable Toxicological Profiling of Small Molecules

**DOI:** 10.1021/acs.jcim.3c00692

**Published:** 2023-09-07

**Authors:** Miriana Di Stefano, Salvatore Galati, Lisa Piazza, Carlotta Granchi, Simone Mancini, Filippo Fratini, Marco Macchia, Giulio Poli, Tiziano Tuccinardi

**Affiliations:** † Department of Pharmacy, University of Pisa, Via Bonanno 6, 56126 Pisa, Italy; ‡ Department of Life Sciences, University of Siena, 53100 Siena, Italy; § Department of Veterinary Sciences, University of Pisa, Viale Delle Piagge 2, 56124 Pisa, Italy

## Abstract

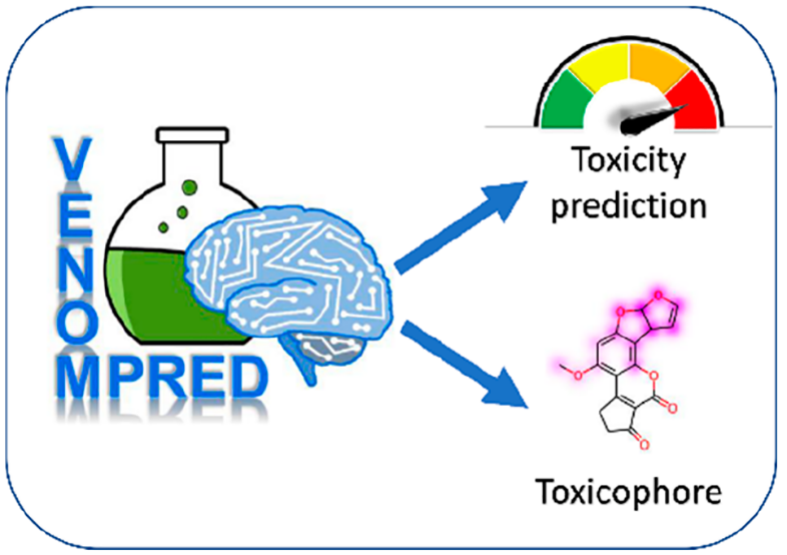

The application of artificial intelligence and machine learning (ML) methods is becoming increasingly popular in computational toxicology and drug design; it is considered as a promising solution for assessing the safety profile of compounds, particularly in lead optimization and ADMET studies, and to meet the principles of the 3Rs, which calls for the replacement, reduction, and refinement of animal testing. In this context, we herein present the development of VenomPred 2.0 (http://www.mmvsl.it/wp/venompred2/), the new and improved version of our free of charge web tool for toxicological predictions, which now represents a powerful web-based platform for multifaceted and human-interpretable *in silico* toxicity profiling of chemicals. VenomPred 2.0 presents an extended set of toxicity endpoints (androgenicity, skin irritation, eye irritation, and acute oral toxicity, in addition to the already available carcinogenicity, mutagenicity, hepatotoxicity, and estrogenicity) that can be evaluated through an exhaustive consensus prediction strategy based on multiple ML models. Moreover, we also implemented a new utility based on the Shapley Additive exPlanations (SHAP) method that allows human interpretable toxicological profiling of small molecules, highlighting the features that strongly contribute to the toxicological predictions in order to derive structural toxicophores.

## Introduction

The application of machine learning (ML) in toxicology, with the aim of developing new artificial intelligence (AI)-based computational models able to predict the toxicity profile of chemicals, is currently taking ground. ML models for *in silico* toxicology are useful to reduce the large costs and long times required by *in vitro* and *in vivo* studies, to limit the ethical issues regarding animal experiments and to allow managing a huge amount of data. In the last years, many efforts have been performed to collect and assess data on multiple properties and hazards of chemical substances; moreover, new guidelines have been applied and shared between private and public companies for regulating and standardizing the use, management and safety protocols of chemical compounds.^1,2^ In this scenario, web tools are becoming a fundamental source of *in silico* toxicological predictions, due to their free availability and the possibility to be used also by unexperienced users.^3^ Therefore, creating a web tool for toxicity predictions is an appealing and compelling strategy to decrease the number of animal tests and to accelerate the development of new drugs. The collaboration between academic and private companies led to the creation of different open-source platforms for sharing data about the toxicity profile of small molecules in a fast and easy way. VEGAHub is one of the most comprehensive online platforms regarding toxicity predictive models and reference data sets.^4^ VEGAHub provides various freely available software, among which VEGA QSAR represents the main one, collecting many different *in silico* methods reported in the literature and implemented therein for predicting toxicity profiles of chemical substances.

In this context, we recently developed ML models able to evaluate the potential mutagenicity, carcinogenicity, estrogenicity and hepatotoxicity of small molecules.^5^ All models were trained and evaluated by using training and test set data derived from VEGA QSAR, which was used as reference software for the performance assessment of our models. The application of a consensus strategy, based on the combination of multiple predictions generated by different ML models, was demonstrated to improve the reliability of the predictions and outperformed all reference models. The consensus ML approach was thus implemented in VenomPred platform, a free of charge web tool for toxicity predictions, able to rapidly generate the probability of mutagenic, carcinogenic, estrogenic, and hepatotoxic effects of small molecules from the SMILES strings of the compounds to be analyzed.

Herein, we report the development of the VenomPred 2.0 platform, which represents a remarkable upgrade of the original web tool. In particular: a) we improved the consensus ML strategy by including a wider combination of ML models, thus further increasing the performance and reliability of all predictions; b) we generated and included consensus ML models for the prediction of four novel toxicity endpoints, namely androgenicity, skin irritation, eye irritation/corrosion, and acute oral toxicity; c) we implemented the SHapley Additive exPlanations (SHAP) method to identify the features that strongly contribute to the toxicological predictions in order to derive structural toxicophores.

The prediction of androgenicity (unwanted androgenic effect) became a crucial point in the early stage of drug development to reduce the risk of failure in later human clinical trials, since several xenobiotics are known to interact with the androgen receptor disrupting normal endocrine functions and consequently causing severe diseases, such as prostate cancer, infertility, androgen insensitivity syndrome and Kennedy’s disease.^6^ Moreover, skin irritation, eye irritation and acute oral toxicity are three toxicity endpoints included in the so-called “6-pack” panel comprising the most widely used animal tests to evaluate the acute toxicity of chemicals.^7^ Therefore, the development of *in silico* models able to efficiently and reliably perform such toxicity evaluations would be strongly desirable in an attempt to reduce the need for *in vivo* experiments. Finally, the need to make ML predictions more transparent, human interpretable, and easily understandable for the whole scientific community, is becoming a hot topic that needs particular attention. In fact, ML models with complicated architectures may achieve high predictive performance but at the cost of a limited interpretability of results, which creates the need for a balance between predictivity and explainability to generate powerful and useful *in silico* approaches.^8^ In this context, there is a growing effort in the development of explainable artificial intelligence (XAI) strategies, able to shrink the gap between computational and experimental chemists and biochemists, and maximize the profitability of *in silico* analyses in drug synthesis planning and safety enhancement.^9^ Among various strategies that can be applied for pursuing this aim, a well-established approach is based on the SHAP (SHapley Additive exPlanations) method,^10^ which is still currently employed for determining the relevance and impact of the input data features (usually molecular descriptors) on ML predictions.^11^ However, the interpretability provided through this method is strongly dependent on the nature of the input features. This aspect may generate a strong limitation, especially in the field of drug discovery and toxicology, where ML models may be trained using complex molecular descriptors that are often devoid of a crystal clear and easily interpretable connection to the structural features of compounds. Therefore, the simple implementation of a SHAP-based analysis in ML models based on molecular descriptors, which enable the evaluation of the impact of the single descriptors on predictions, generate information that is certainly useful for chemoinformatics but have poor relevance for medicinal chemists and biochemists seeking for clear guidelines for decision-making processes (such as synthesis planning or compounds selection) based on *in silico* toxicological evaluations. What is actually useful for the scientific community is the possibility to interpret and decipher the predictions of ML models from a structural point of view and directly visualize which moieties of the analyzed molecules may have toxic/unwanted features. For this reason, we are recently observing both a tendency to generate predictive models based on more easily interpretable molecular representations, such as substructural molecular fingerprints, and the development of approaches able to decode atom and nonatom attributions representing them directly on molecular structures, for a direct and easy interpretation of predictions.^9,12^ Inspired by these recent and valuable approaches, we developed a robust SHAP-based explainability protocol with automatic attribution-to-structure decoding (retro-mapping), able to directly identify the specific molecular fragments and moieties with the highest impact on the ML predictions and thus responsible for potential toxicological effects of small molecules. Our approach, which ensures a crystal clear interpretation of *in silico* predictions and can thus be easily exploited by the whole scientific community, may not only contribute to rationalize specific structural-toxicity relationships but also provide invaluable help for the design of safer compounds and for further limiting the need for *in vivo* experiments. All these features are now available on VenomPred 2.0 and freely accessible at http://www.mmvsl.it/wp/venompred2.

## Materials and Methods

### Modeling Data Sets

For mutagenicity, carcinogenicity, hepatotoxicity, and estrogenicity endpoints, the same training and test sets employed in our previous work were used (Table S1). The data set used for the generation of the androgenicity models was collected from ToxCast/Tox21 and ChEMBL databases, which include molecules with associated biological data evaluated with three different *in vivo* assays: competitive binding assay, receptor gene assay, cytotoxicity assay.^13−15^ The retrieved compounds were classified according to the criteria applied by Tan and co-workers.^16^ Specifically, a compound was labeled as toxic if it showed binding activity in at least one competitive binding assay and if the activity was detected in at least one reporter gene assay. Compounds were labeled as nontoxic if the binding assay and all reported gene assays yielded negative results. The data sets for skin irritation, eye irritation and acute oral toxicity endpoint were collected from literature and publicly available databases, and then curated and classified according to the protocol used by Borba et al.^17^ The data set for skin irritation was obtained from the publicly available REACH studies,^18^ and compounds were classified as toxic if they exhibited irritant or corrosive properties. Concerning irritation, compounds were considered as toxic if their corresponding average erythema/edema score was higher than 2.3, as reported in the Globally Harmonized System of Classification and Labeling of Chemicals (GHS).^19^ In terms of corrosivity, a compound was classified as toxic if irreversible skin irritation and corrosion effects were reported. Eye irritation data were collected from the REACH database and several additional literature sources,^20−27^ and were classified according to the results obtained after application of a single dose of the chemical. A compound was considered as toxic if a damaging effect on the conjunctiva, cornea, and iris was reported. For the acute oral toxicity endpoint, the data set was obtained from the Interagency Coordinating Committee on the Validation of Alternative Methods (ICCVAM).^28,29^ The compounds within the data set were classified taking into account their corresponding lethal dose (LD_50_). Specifically, compounds with an LD_50_ value lower than 2000 mg/kg were considered as toxic. For each endpoint, the initial data set was processed to remove inconsistent and duplicate instances; then, the structures of the compounds were represented with standardized SMILES. Finally, the refined data set obtained for each endpoint was divided into a training set and a test set, respectively including 80% and 20% of the original data set, by using a random-splitting strategy. The final composition of the data sets used for the generation and evaluation of all developed ML models for the four new endpoints is shown in Table 1.

**Table 1:** Total Number of Molecules Present in Training and Test Sets Employed for Generating Androgenicity, Skin Irritation, Eye Irritation, and Acute Oral Toxicity Models

Androgenicity Model			
Data Set	Total	Nontoxic	Toxic
Training	2274	1592	682
Test	568	394	174

Skin Irritation Model			
Data Set	Total	Nontoxic	Toxic
Training	810	554	256
Test	202	141	61

Eye Irritation Model			
Data Set	Total	Nontoxic	Toxic
Training	2836	1925	911
Test	709	475	234

Acute Oral Toxicity Model			
Data Set	Total	Nontoxic	Toxic
Training	6754	2901	3853
Test	1688	738	950

The training and test sets obtained for each endpoint were subjected to dimensionality reduction using the t-distributed stochastic neighbor embedding (t-SNE) algorithm, applied to the compounds encoded as PubChem FPs. The analysis showed that training and test set compounds of each endpoint are properly overlaid and cover a comparable chemical space (Figure S1), thus confirming that each test set properly represents the corresponding training set to be used for model development.

### Molecular Fingerprints

For each data set, molecular representations of chemical compounds were calculated. Specifically, based on the results of our previous work, Morgan, RDKit and PubChem chemical fingerprints (FPs) were computed. Morgan and RDKit FPs were generated with the RDKit python library,^30^ while PubChem FPs were calculated with the PyBioMed python module.

*Morgan FPs* represent the structure of compounds based on the surrounding atoms and bonds within a certain distance between atoms, (which was set to 2 in this work) and assigning them a unique identifier.^31^ These identifiers are then usually hashed to a bit vector with a fixed length in order to allow the comparison of different representations. In this work, we set a vector length of 1024 bits using the Morgan FPs implementation of RDKit.

*RDKit FPs* are RDKit-specific fingerprints inspired by public descriptions of the Daylight topological fingerprints. The fingerprinting algorithm identifies all subgraphs in the molecule within a particular range of sizes, hashes each subgraph to generate a raw bit ID, adjusts the raw bit ID to fit in the assigned fingerprint size, and then sets the corresponding bit.

*PubChem FPs* consist of substructure-based FPs represented with a vector of 881 bits, in which each bit encodes the presence of an element or substructure, a specific ring system, the atom pairs and the atom’s nearest neighbors.^32^

### Classification Models

The ML models generated for the prediction of the different toxicity endpoints were developed using four different classification algorithms: random forest, support vector machine, k-nearest neighbor, and multilayer perceptron. The dedicated functions of the python Scikit-learn^33^ library were used to generate the models (more details about Scikit-learn library are reported within Supporting Information).

#### Random Forest (RF)

The algorithm consists of a large number of decision trees that work as an ensemble. Each individual tree provides a class prediction.^34^ The class that obtains the majority of votes represents the final prediction of the model. The main hyperparameters optimized during model construction were *max_features*, which expresses the maximum number of features that can be considered in a single tree, and *n_estimators*, which indicates the number of trees constructed before the predictions. The options evaluated as *max_features* were (a) *sqrt*, which is the square root of the total number of features in a single node; (b) *log2*, which corresponds to the binary logarithm of the total number of features for a single node; and (c) *None*, for which *max_features* corresponds to the total number of features. The number of estimators that were considered corresponds to 100 and 500.

#### Support Vector Machine (SVM)

SVM maps the data according to their common patterns and aims toward their optimal division between two classes, with each of them entirely lying on opposite sides of a separating hyperplane. The aim is achieved by maximizing the distance between the closest training data points, the so-called support vectors, and the hyperplane.^35^ The hyperparameters optimized during model building were: a) the *kernel*, which represents the function to map the data into a higher dimensional feature space in order to make them separable; and b) *C*, which indicates how much emphasis is made on the misclassified data, therefore helping in optimizing the hyperplane.

#### k-Nearest Neighbor (KNN)

The KNN algorithm categorizes instances based on the classes of their neighbors. The class of an instance is predicted by considering the most represented class among its *k* nearest neighbors. The final prediction is therefore obtained by the most frequent output among the features of the nearest neighbors to the input data.^36^ The hyperparameters optimized during model generation were those that reduce the error due to the voting of the surrounding neighbors,^37^ namely *n_neighbors* and *weight*. *n_neighbors* represents the number of neighbors taken into account for the classification, whereas *weight* defines how much the different surrounding elements affect the final prediction. The values investigated for *n_neighbors* were in a range between 1 and 15, while two options were evaluated for *weight*: a) *uniform*, indicating that all points in each neighborhood are equally weighted, and b) *distance*, imposing that closer neighbors of a query point have a higher influence than neighbors that are farther from it.

#### Multilayer Perceptron (MLP)

MLP is a type of feedforward artificial neural network (ANN) composed by multiple layers of nodes, which uses a supervised learning strategy called backpropagation.^38^ Four hyperparameters were tuned in order to minimize the error of the output predictions: a) *hidden_layer_size*, which indicates the number of neurons and the number of hidden layers; b) *solver*, which is a fundamental parameter to optimize the predictions at every decision step through the different layers; c) *activation*, which refers to the activation function and defines how the weighted sum of the input is transformed into an output by one or more nodes in each network layer; and d) *learning_rate_init*, which controls the step-size in updating the weights. For the *hidden_layer_size*, we evaluated all possible combinations of a set of 100, 200, and 1000 neurons. The *solvers* tested were: *lbfgs*, which uses a limited amount of computer memory, only storing a certain number of vectors, as well as the stochastic gradients *adam* and *sgd*. Among the activation functions, we considered *identity*, *logistic*, *tanh, and relu* functions. The options investigated for *learning_rate_init* were 0.01, 0.001, and 0.0001.

### Model Building and Evaluation

By employing 3 different chemical FPs and 4 different ML algorithms, 12 different toxicity models were generated for each endpoint considered. An optimization process based on Grid Search cross-validation implemented within Scikit-learn was applied to all generated models for tuning the best hyperparameters setting on training data set (more details about Scikit-learn library are reported within the Supporting Information).^39^ In particular, the Grid Search cross-validation consisted of dividing the training set into several subsets or folds and iteratively training and evaluating the model on different combinations of these folds. The main objective of cross-validation was to estimate the performance of the model on unseen data by exhaustively evaluating all possible combinations of hyperparameter values, assigning a score to each of them. In this work, the scoring parameter used was Matthew’s correlation coefficient (see the next section for details).

### Final Model Evaluation Metrics

To assess the performance of the 12 optimized models obtained for each endpoint, a validation was performed using the test sets initially generated during the data set processing step (Table 1). The predictive performance of the models was evaluated by taking into account five statistical parameters: precision, specificity, recall (or sensitivity), accuracy, and Matthew’s correlation coefficient (MCC), which are defined as follows:
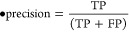

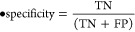

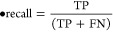

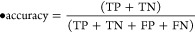

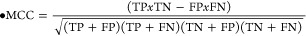
TP (true positives) and TN (true negatives) indicate the number of toxic and nontoxic compounds, respectively, correctly predicted as such; FP (false positives) represents the number of nontoxic compounds predicted as toxic and FN (false negatives) indicates the number of toxic compounds predicted as nontoxic. Therefore, precision measures the ability of the model to provide positive predictions by quantifying the rate of correct positive predictions over the total positive predictions, also including false positives, while specificity and recall measure the ability of models to correctly predict negative and positive instances, respectively. Accuracy and MCC take into account all of the values derived from binary classification. Accuracy is simply the rate of correct predictions over all predictions, while MCC represents a more balanced evaluation of the classifier performance.^40^ For instance, MCC = 1 indicates a perfect classification (with no FP and FN), MCC = 0 is equivalent to random classification, and MCC = −1 indicates a complete disagreement between predicted and actual classes. In parallel, a y-randomization test was performed for each of the 12 optimized models obtained for each endpoint. The analysis strongly confirmed the reliability of our models. In fact, as expected, MCC values of zero and accuracy values around 0.50, indicating completely random predictions, were obtained as a result of the randomization tests (see Supporting Information for details).

### Consensus Strategy and Consensus Score

The consensus approach consisted in generating the final toxicity prediction of a molecule for a specific endpoint (called consensus prediction) from the combination of the predictions provided by multiple models for such endpoint. In detail, when a ML model returns a toxicity prediction for a given compound, a probability score (PS) in the range from 0 to 1 (0 ≤ PS < 0.5 if predicted as nontoxic; 0.5 ≤ PS ≤ 1 if predicted as toxic) is also provided. By following the consensus approach, a consensus score (CS) was generated by averaging the PSs produced by each model included in the consensus prediction; a compound was then labeled as nontoxic by the consensus prediction if the obtained CS was less than 0.5 and toxic if the CS was equal to or greater than 0.5. In order to exhaustively evaluate all possible consensus predictions that could be generated for each molecule by combining the results of the single ML models, all possible combinations of models were analyzed. Since 12 different optimized models were developed for each endpoint, we calculated all possible combinations of predictions considering permutations in the range of 2–12, by applying the following combinatorial formula:
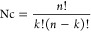
1where *n* corresponds to the total number of models (12) and *k* varies according to the number of permutations allowed (2–12). Finally, by summing the results of each permutation obtained for each endpoint using the described formula, we derived the total number of unique combinations (Nc), which was found to be 4083. The same statistical parameters used to evaluate the performance of the single optimized models (precision, recall, specificity, MCC, and accuracy) were then calculated based on the consensus predictions to evaluate the performance of the consensus strategy. The predictive performance in terms of MCC of the best consensus combination identified for each endpoint was also evaluated in relation to a possible applicability domain of our models. The analysis confirmed the high reliability of our consensus strategy (see Supporting Information for details).

### Feature Contributions

Feature contributions to model predictions were calculated by following the Shapley value approach. The SHAP (SHapley Additive exPlanations) method was originally introduced to estimate the importance of an individual player in a collaborative team.^10^ With this approach, the impact of the team members was evaluated, taking into account their individual contributions to the final outcome of a game. Shapley values proved to be a robust approach for fair and reasonable evaluation of each individual’s importance by obtaining a unique result characterized by the following axioms: local accuracy, consistency and null effect.^41^ The idea behind the use of SHAP values to explain ML models is based on the identification of important features that are directly related to the outcome of the model.^42^ Focusing on binary classifiers such as the models developed herein, the importance of features was provided with a sign that corresponds to the direction of the magnitude of contribution. Specifically, the positive sign identifies a contribution to the prediction of toxicity, while the negative sign results in a contribution to the prediction of nontoxicity. Given the model-dependent nature of the SHAP approach, we decided to employ the Kernel SHAP model-agnostic method provided by the SHAP python library. Kernel SHAP is based on an extension of LIME,^43^ a local approximation approach to rationalize model decisions. Since the computational costs associated with the exact computation of all Shapley values are considerable, such a local approximation approach represents a valid alternative for obtaining reliable explanations of machine learning models. The feature contribution analysis was not performed during cross-validation but was made available in VenomPred 2.0 for the analysis of new compounds, thus allowing the users to visualize which moieties of the tested molecules may have toxic/unwanted features, so that they can profitably exploit this information (e.g., planning an optimization of the molecules aimed at replacing the potentially toxicophoric groups with unharmful moieties).

### Feature Importance Mapping

The Kernel SHAP method was applied for each toxicity endpoint to calculate the feature importance from the models that formed the best consensus approach. In order to generate a rational visualization of the results obtained from the SHAP analysis, the weights of the atoms were calculated using a bit retro-mapping approach. Specifically, since three different chemical fingerprints were used to represent the structure of the compounds, an appropriate bit retro-mapping method was applied for each of them. For Morgan and RDKit FPs, the built-in functions of the RDKit library were used to identify the atoms that contributed to each on-bit. An in-house protocol was instead applied to match the atoms of the PubChem FPs. Since the latter molecular representation belongs to substructure-based FPs, direct associations between the query and matching atoms were retrieved by substructure matching. SMARTS queries were obtained from the official PubChem fingerprints documentation. Nevertheless, this approach revealed a problem for matching bits in the range between 115 and 262, since no SMARTS structure was reported for them. In particular, this group of bits encodes the presence and occurrence of the number of different ring systems described in the documentation. Given the redundant nature of this group of bits, we decided to apply a protocol for a rational mapping of these bits in order to avoid potentially redundant (and thus biased) atom correspondences. The approach consists of clustering the bits that encode for either the same or overlapping ring systems and only differ in the count of occurrences. Finally, for each cluster of bits, only the highest corresponding feature score was retained.

Following the protocols described above, atoms corresponding to individual FP bits were identified for each compound and then a feature weighting method was applied to assign a reliable score to each identified atom.^44^ Specifically, the weight of each atom for a given molecule (fw) was calculated by dividing the score of each feature that included such an atom by the number of associated atoms (*n*_Atoms_), scaled by the number of occurrences of the feature (*n*_occ_):
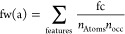
The final atom weight was defined as the average weight of each atom obtained from each model belonging to the best consensus approach. Finally, the atom weights were visualized by using the mapping functions of RDKit.

## Results and Discussion

### Model Generation and Optimization

The development of VenomPred 2.0 platform, an update of the original web tool, was born from the idea of expanding the pool of toxicological endpoints evaluated by the platform and providing optimized models with maximized performance. Moreover, we aimed to implement the SHAP method to understand which structural features contributed to the prediction of toxicity. These features provide insights into the explainability of the models, allowing us to visualize structural moieties with high toxicological impact. The new toxicity endpoints that were analyzed are androgenicity, skin irritation, eye irritation, and acute oral toxicity. The choice to develop models for androgenicity predictions, in addition to our recently developed estrogenicity models, stems from the growing interest in recent years in identifying endocrine disrupting chemicals (EDCs), whose mechanism of action is still unknown. EDCs can interact with nuclear receptors, including estrogen receptor α (ERα) and androgen receptor (AR), to impair the physiological functions of the endocrine system, causing severe symptoms.^45,46^ The ToxCast project of the U.S. Environmental Protection Agency (U.S. EPA) revealed that 12.2% and 8.4% of the chemicals examined cause damages through interactions^47^ and/or modulation of ERα and AR, respectively. Moreover, we focused on analyzing endpoints whose evaluation is still conducted with *in vivo* assays, thus not satisfying the call to reduce, refine, and replace (the three Rs) animal tests for hazard identification.^48^ Indeed, skin irritation, eye irritation, and acute oral toxicity are part of the so-called “6-pack” panel of tests,^7^ the most common type of animal tests employed for assessing the acute toxicity of chemicals such as pesticides, pharmaceuticals or cosmetics. Several sources were used to extract the data sets for generating our toxicity models. The collected data sets were then subjected to a refinement phase that allowed the removal of duplicate instances and the creation of training and test sets. Detailed information about the composition of these data sets is reported in the Materials and Methods section (Table 1).

**Figure 1:**
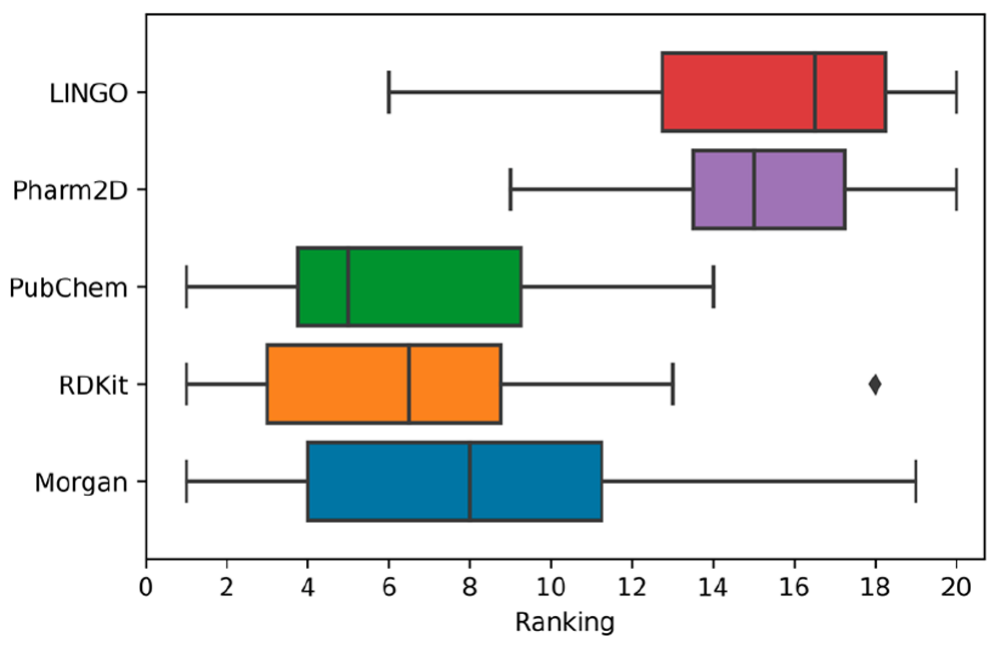
Ranking distribution in terms of MCC for the five groups of models based on the same FP type previously generated for the development of VenomPred.

With the aim of obtaining robust and reliable toxicity predictions for the four new endpoints, we planned to employ a consensus approach, a strategy that already proved successful in docking and virtual screening studies,^49−52^ as well as in ML-based toxicity predictions, as we recently demonstrated.^5^ In this context, the consensus strategy relies on the use of multiple ML models whose predictions are combined together for providing the final consensus prediction of each molecule. In particular, a single ML model labels a molecule as either toxic or nontoxic based on the predicted probability score (PS). If the PS predicted for a molecule is below 0.5, the molecule is labeled as nontoxic; otherwise (0.5 ≤ PS ≤ 1), it is labeled as toxic. Analogously, the consensus prediction of a molecule is based on the consensus score (CS), which corresponds to the average of the different PSs obtained as output from each individual model included in the consensus approach. If the CS predicted for a molecule is below 0.5, the molecule is labeled as nontoxic; otherwise (0.5 ≤ CS ≤ 1), it is labeled as toxic. This strategy was already successfully applied for the development of VenomPred platform, in which we employed a consensus of the 5 top-scored ML models out of the 20 different models generated combining four different ML algorithms and five molecular fingerprints (FPs). In the present work, we aimed at maximizing the power of the consensus strategy by performing an exhaustive consensus analysis, evaluating the reliability of as many combinations of ML models as possible to identify the most reliable one. However, we envisioned that employing 20 different models a total of 1,048,555 consensus combinations were possible. Due to the huge amount of time that would be necessary to evaluate all such combinations, we planned to generate a lower number of highly reliable ML models to be combined in the extensive consensus analysis. For this reason, we carried out a performance analysis of the models generated in the development of VenomPred and observed that the ML models based on PubChem, RDKit and Morgan FPs performed statistically better than the other ones, which employed LINGO and Pharm2D FPs. Figure 1 shows the ranking distribution in terms of the Matthew’s Correlation Coefficient (MCC) of the five groups of models based on the same FP type. As shown in the figure, the median ranking in terms of MCC obtained by models based on PubChem, RDKit and Morgan FPs is significantly higher that achieved by the other two groups of models. In particular, the distribution shows that about 75% of models based on PubChem, RDKit and Morgan FPs have a rank not lower than 11 in terms of MCC, while about 75% of models based on LINGO and Pharm2D FPs have a rank higher than 13; therefore, the average performance of the models based on PubChem, RDKit and Morgan FPs appears to be substantially higher than that of the others.

**Figure 2:**
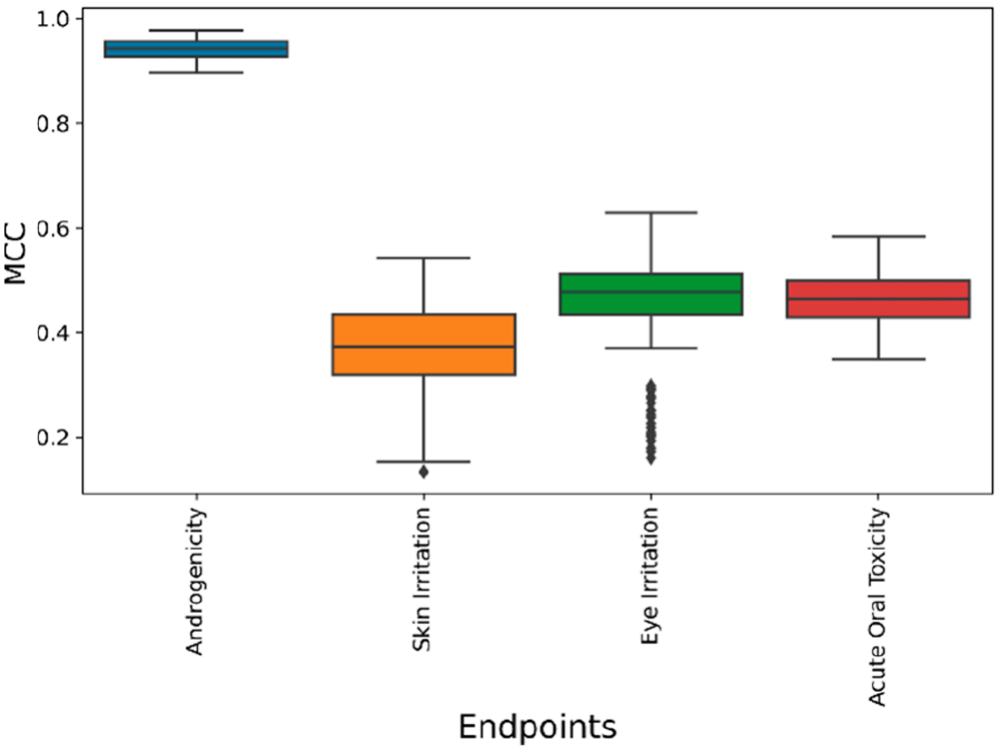
Cross-validation results, expressed in terms of MCC, obtained for all different models developed for the androgenicity, skin irritation, eye irritation, and acute oral toxicity endpoints.

According to this data, we only considered Morgan, RDKit and PubChem FPs for generating the models for the four new endpoints and for applying the exhaustive consensus analysis. Therefore, the training set compounds of the four new endpoints were represented as SMILES strings and then translated into the three considered types of FPs. Then, each FP type was combined with four different classification algorithms, i.e., random forest (RF), support vector machine (SVM), k-nearest neighbor (KNN) and multilayer perceptron (MLP), which led to the development of 12 different classification models for each endpoint, a much more manageable ensemble of ML models for performing the exhaustive consensus analysis (*vide infra*). The hyperparameters of the models were tuned on the training set through a combined GridSearch (see Supporting Information for details) and cross-validation approach. Cross-validation (CV) helps estimate the performance of the models. It is useful for model evaluation and optimization, for adjusting hyperparameters, and for checking the performance of the models with different subsets of the available data. In this work, we conducted a 20-fold random-split CV, which means that we randomly split 80/20 the available data for 20 folds. Precisely, in each fold, the models were trained with 80% of data (CV training set), whereas the remaining 20% (CV test set) was used to assess the performance of the models. Therefore, for each endpoint we obtained 20 different sets of predictions that allowed the calculation of 20 different performance estimations for each generated model. The performances were evaluated in terms of MCC, which represents a reliable balanced index for the evaluation of the classifier performance (see Materials and Methods for details). The results are summarized in Figure 2. Overall, the results highlight that the androgenicity endpoint obtained an outstanding predictive performance in terms of MCC (above 0.90), while for the other endpoint less impressive but still widely satisfying results were obtained. In particular, for the eye irritation and acute oral toxicity endpoints we obtained MCC values around 0.50, whereas the skin irritation predictive models showed MCC values around 0.4. These results are fully consistent with the performance evaluation results based on the test set prediction (*vide infra*). The best combination of hyperparameters was then selected based on the best results obtained in terms of MCC.

### Model Evaluation and Consensus Strategy

All developed models for each new endpoint were then subjected to a final evaluation consisting of predicting the potential toxicity of the test set molecules, which were not used for model training. The performance of the 12 models built for each endpoint was calculated in terms of recall, precision and specificity, besides accuracy and MCC (see Materials and Methods for details). Table 2 shows the performance achieved by the top-scored model obtained for each new endpoint in terms of MCC, precision, recall and specificity. For all endpoints, the models based on RF and MLP algorithms combined with PubChem FPs achieved the best performance. Therefore, RF and MLP were found to be the most powerful algorithms, while PubChem FPs were the most effective FPs to distinguish between toxic and unharmful compounds in relation to these four endpoints. In particular, androgenicity was the endpoint for which the highest predictive efficacies were obtained, with almost ideal values of precision, specificity and accuracy (Table 2). In fact, the whole set of models generated and optimized for this endpoint showed outstanding results, with MCC values equal to or above 0.90 (Figure S2A). The 12 models generated for the acute oral toxicity endpoint showed an average MCC value around 0.50 (Figure S2D), with the best model achieving a value of 0.55 (Table 2), which indicates that more than 75% of the toxicological predictions performed on the test set compounds were correct, as also confirmed by the accuracy value of 0.78. A satisfying performance was also obtained for the best models of the other two endpoints. The models generated for toxicological predictions of eye and skin irritation showed average MCC values around 0.40 (Figure S2), reaching a maximum of 0.44 and 0.49, respectively, with the two best models based on the MLP algorithm. Nevertheless, the reliability of their performance was confirmed by the precision values, indicating that at least 65% of the compounds predicted as toxic were correctly labeled, as well as by the specificity scores above 0.80, indicating a high reliability in the prediction of unharmful compounds. In fact, both models showed accuracy values above 0.75, which confirmed that more than 75% of the test set predictions were correct (Table 2).

**Table 2:** Performance Evaluation Results, Based on Test Set Prediction, Obtained for the Top-Scored Models in Terms of MCC for Androgenicity, Eye Irritation, Skin Irritation, and Acute Oral Toxicity Endpoints

Endpoint	Model	MCC	Precision	Recall	Specificity	Accuracy
Androgenicity	RF_PubChem	0.94	0.99	0.93	0.99	0.98
Eye Irritation	MLP_PubChem	0.44	0.65	0.59	0.84	0.76
Skin Irritation	MLP_PubChem	0.49	0.67	0.61	0.87	0.79
Acute Oral Toxicity	RF_PubChem	0.55	0.79	0.82	0.73	0.78

**Figure 3:**
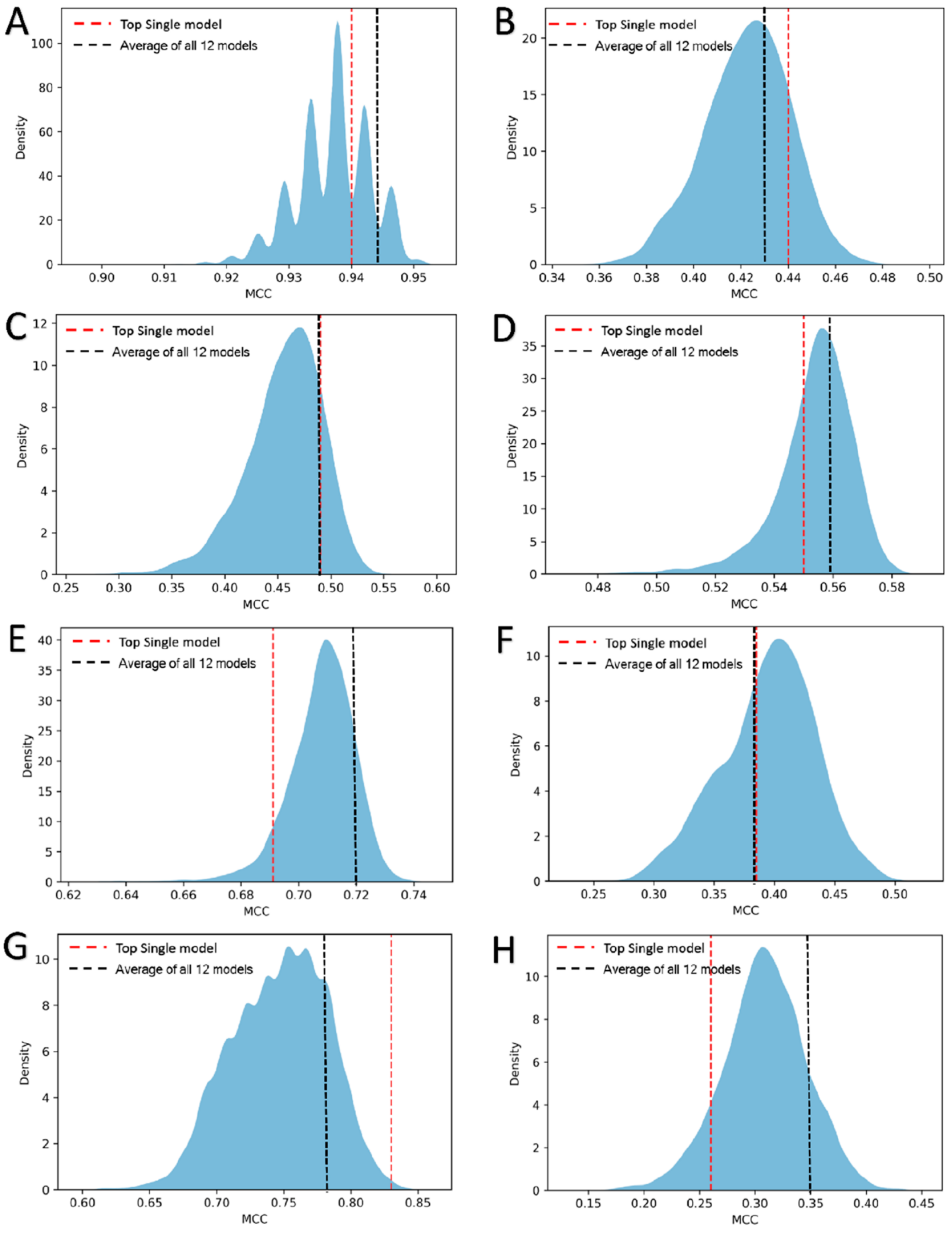
Distribution of performance in terms of MCC obtained for the prediction of the test set compounds using different combinations of models. In each plot the performance achieved using the predictions of the top-scored single model (red dashed line) and by averaging the predictions of all 12 models used for the analysis (black dashed line) is shown as a reference for A) androgenicity, B) eye irritation, C) skin irritation, and D) acute oral toxicity endpoints. E) mutagenicity, F) carcinogenicity, G) estrogenicity, and H) hepatotoxicity endpoints.

In an attempt to improve the predictive performance obtainable with the use of the individual models, we applied exhaustive consensus analysis. In particular, given the 12 models developed for each endpoint, we calculated all possible combinations of predictions, considering the PSs associated with the toxicity predictions provided by each model for each tested compound. Finally, we calculated the CS by averaging the PSs associated with the toxicity predictions provided by each selected model. By performing this exhaustive analysis with 12 different ML models, a total of “only” 4038 combinations for each endpoint were tested, in spite of more than a million combinations that would have originated from 20 different models. This novel exhaustive analysis was also applied to the 12 ML models based on Morgan, RDKit and PubChem FPs developed for mutagenicity, carcinogenicity, hepatotoxicity, and estrogenicity endpoints in the first version of VenomPred, which were previously analyzed with a nonexhaustive consensus strategy. Figure 3 shows the distribution of results in terms of MCC obtained by predicting the test set compounds with the 4038 different consensus combinations for each of the eight total endpoints considered. The different combinations of models showed a nearly normal distribution of performance for each endpoint. However, the variance of the distribution was found to be rather different. For half of the endpoints considered, namely, skin irritation, carcinogenicity, estrogenicity, and hepatotoxicity, we observed a remarkable difference between the lowest and highest MCC values achieved by the different consensus combinations. For instance, MCC values ranging from 0.15 to 0.43 were obtained for the hepatotoxicity endpoint (Figure 3H), while skin irritation endpoint showed a range of MCC values from 0.28 to 0.58 (Figure 3C). On the contrary, the androgenicity endpoint (Figure 3A) showed a much shorter range of MCC values (between 0.90 and 0.95), whereas intermediate results were obtained for the remaining endpoints. It is thus evident that different combinations of ML models can produce significantly different results, and it is desirable to evaluate the performance of many different combinations with the aim of maximizing the reliability of the consensus approach. Overall, our analysis confirmed that the consensus strategy is able to improve the statistical performance of toxicological predictions, since for all endpoints an appreciable increase in terms of MCC values was obtained using the best consensus combination, compared to the single top-scored model. Moreover, the analysis highlighted that the consensus strategy appears to be particularly suitable to succeed in achieving reliable predictions in those cases in which the single models alone do not reach extremely satisfying results. In fact, the highest improvements in terms of MCC with respect to the best single models were obtained for carcinogenicity (Figure 3F) and hepatotoxicity (Figure 3H) endpoints. Precisely, an increase in MCC from 0.39 to 0.50 was obtained for the carcinogenicity endpoint using a seven-model consensus approach (Tables S2 and S3), which produced a considerable increase also in terms of Precision (from 0.73 to 0.80) and Specificity (from 0.67 to 0.76). For the hepatotoxicity endpoint, the consensus analysis identified a 4-model consensus combination that achieved an MCC of 0.43 (Table S3) compared to the value of 0.26 showed by the best single model (Table S2); in particular, a remarkable improvement in terms of recall (from 0.86 to 0.97) was obtained using the consensus predictions. Considerable although less impressive performance improvements were also obtained for the other endpoints. For instance, an increase in terms of MCC values from 0.49 to 0.58 were obtained for the skin irritation endpoint using a consensus of 4 different models (Tables 2 and S3), which allowed to boost all statistical parameters considered and, in particular, Precision (from 0.67 to 0.73) and Recall (0.61 to 0.67). Similar improvements were observed for eye irritation, acute oral toxicity, and mutagenicity endpoints (Tables 2, S2 and S3). The two endpoints for which only minor performance gains were achieved using the consensus strategy were androgenicity (Figure 3A) and estrogenicity (Figure 3G), whose single top-scored models showed MCC values (0.94 and 0.83, respectively) comparable to those obtained using the consensus approach (0.95 and 0.84, respectively, Table S3). Nevertheless, the 6-model consensus combination of the androgenicity endpoint (Table S3) allowed to reach ideal values of precision and specificity (1.00), while the 3-model consensus selected for the estrogenicity endpoint (Table S3) showed a better balance of precision, recall and specificity (0.90, 0.91 and 0.93, respectively) compared to the best single model (precision = 0.92, recall = 0.85, specificity = 0.96).

In conclusion, the exhaustive consensus analysis allowed us to obtain optimized predictive strategies for all the different endpoints considered, which were able to generate a minimum of 73% (for the hepatotoxicity endpoint) up to a maximum of 98% (for the androgenicity endpoint) of correct predictions, as shown by the Accuracy values obtained (Table S3). Moreover, for five out of the eight endpoints considered, a percentage of correct predictions above or equal to 80% was obtained (Table S3), thus confirming the high reliability of our predictive strategies. On the contrary, the simple average of the predictions obtained by all 12 different models employed for the exhaustive consensus analysis, which represents one out of the 4083 combinations tested, did not show suitable results. In fact, for five out the eight endpoints considered, i.e., androgenicity (Figure 3A), eye irritation (Figure 3B), skin irritation (Figure 3C), acute oral toxicity (Figure 3D), and carcinogenicity (Figure 3F), the simple averaged predictions of the 12 models produced MCC values equal to or comparable to those generated by the top single model of such endpoints. Therefore, for most of the endpoints considered, the combined use of all 12 models represents a rather useless strategy, as it generates the same predictive performance spending a much higher computation time, requiring the prediction of 12 different models instead of that of a single model. Only for mutagenicity (Figure 3E) and hepatotoxicity (Figure 3H) endpoints, this strategy produced better performances in terms of MCC than those generated by the top single models. Nevertheless, such performances were still weaker than those obtained by using the best consensus strategies identified by the exhaustive analysis (especially for the hepatotoxicity endpoint) and were also less efficient in terms of computation time, requiring the predictions of 12 models instead of only 6 and 4 models, respectively (Table S3). Finally, for the estrogenicity endpoint (Figure 3G) the averaged predictions of all 12 models produced results statistically worse than those generated by the top single model. This last analysis further confirms the reliability of our exhaustive consensus evaluation and the importance of testing all possible combinations of models for each endpoint for identifying the best predictive strategy.

**Figure 4:**
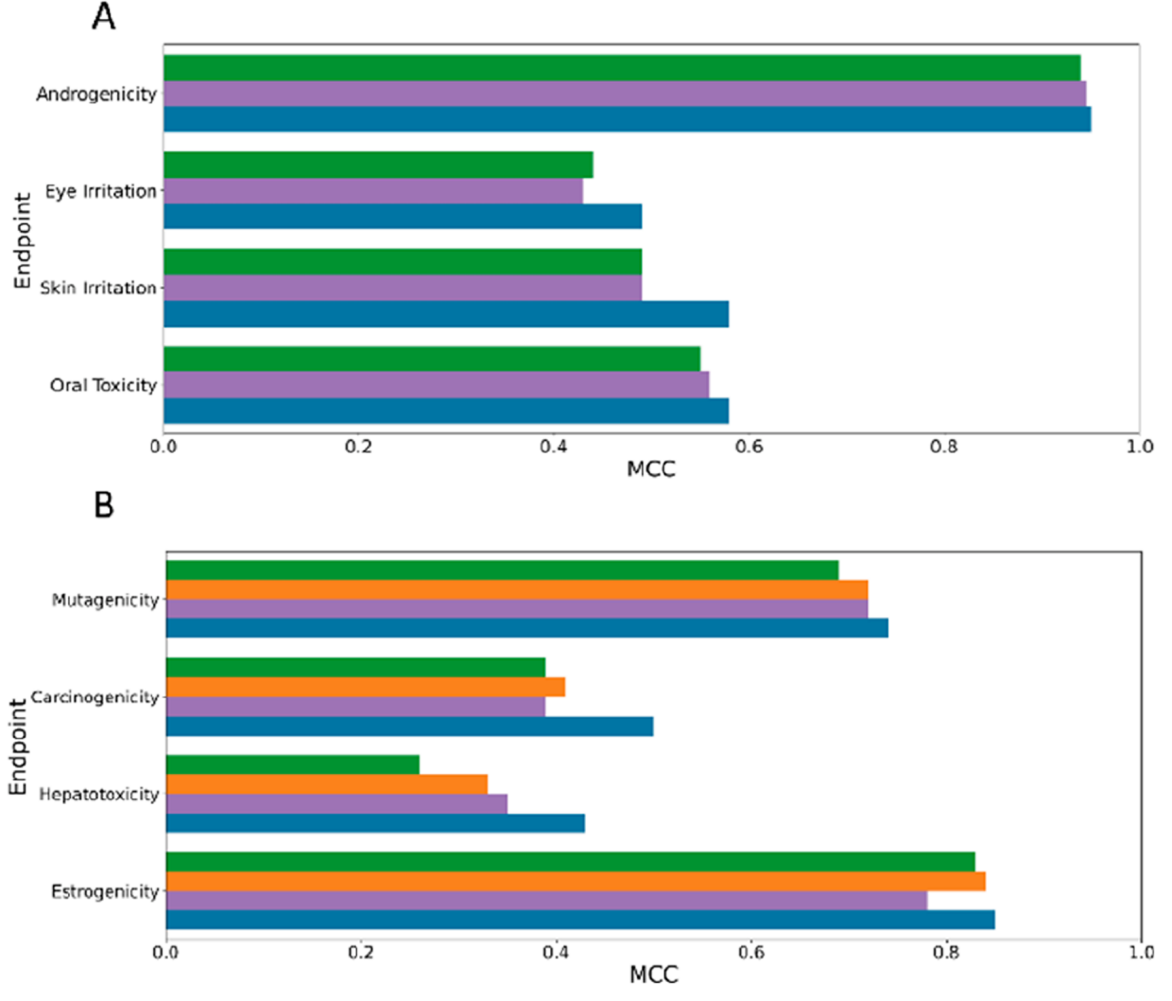
Performance of the best consensus prediction strategy obtained for the new (A) and previously studied (B) endpoints, based on the novel exhaustive analysis. The MCC values obtained using such consensus predictions are shown in blue, green bars indicate the MCC values obtained using the best single ML models of all endpoints herein considered, whereas purple bars indicate the MCC values obtained averaging the predictions of all 12 models used for the exhaustive consensus analysis. In panel B, orange bars indicate the MCC values obtained using the old consensus strategy considered in our previous work.

Figure 4 summarizes the final results obtained by applying the exhaustive consensus analysis for the eight toxicity endpoints. In particular, for androgenicity, eye irritation, skin irritation and acute oral toxicity endpoints, the increase in terms of MCC achieved by the best consensus combination with respect to the best single models is shown (Figure 4A), whereas for mutagenicity, carcinogenicity, hepatotoxicity and estrogenicity, the results of the exhaustive consensus approach are also compared with those previously obtained with the nonexhaustive consensus analysis (Figure 4B).^5^ Finally, for all the endpoints considered, the performance obtained by averaging the predictions of all 12 models used for the analysis is also shown (Figure 4). The results displayed in Figure 4 further remark on the reliability of the exhaustive consensus analysis herein performed. In fact, for the latter group of four endpoints, our study led to identify combinations of ML models that not only performed better than the single top-scored models, but also showed improved predictive performance with respect to the consensus combinations identified in our previous work through a nonexhaustive analysis. Moreover, the best increase in terms of MCC values obtained with the novel consensus predictions herein identified, with respect to the old ones, were obtained for the carcinogenity and hepatotoxicity endpoints, for which the single ML models showed a lower performance compared to the other endpoints. This further supports the hypothesis that the exhaustive consensus strategy can be particularly useful for improving the reliability of toxicological predictions in those cases in which a boost in predictive performance is most needed. Additionally, the results herein shown suggest that the application of a more simplistic consensus approach, in which the predictions of all single ML models developed are averaged, is generally not proficient in terms of efficiency and (in most cases) quality of predictions, and further confirms the importance of an exhaustive consensus analysis for achieving the best possible performance improvement.

### Importance of Features

**Figure 5:**
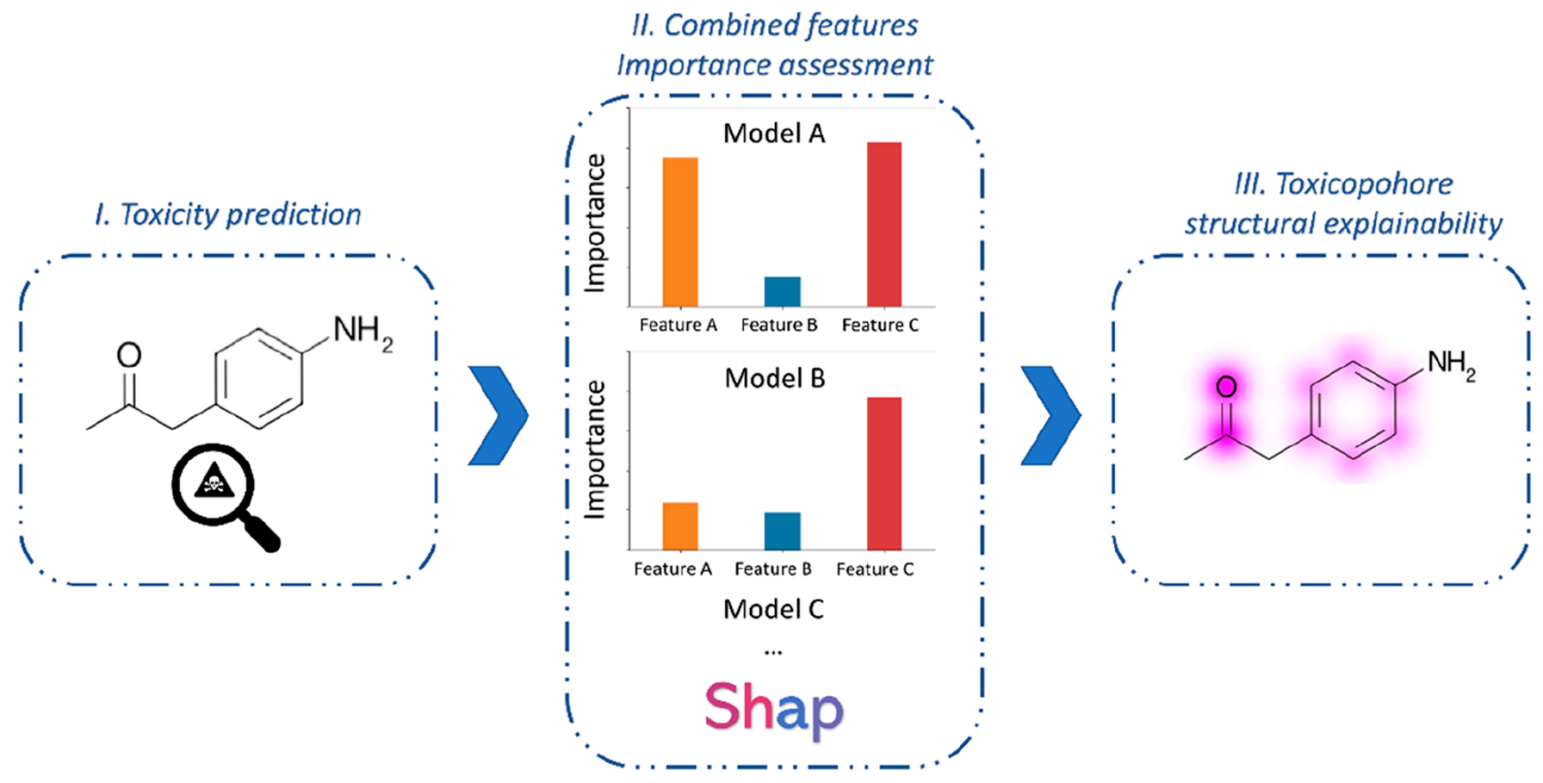
SHAP method workflow: (I) Toxicity prediction; (II) Assessing features importance obtained from the models of the best consensus combination; (III) Retro-mapping of feature impact to highlight the moieties that strongly influence the toxicity prediction.

Once we identified the best consensus approach for each of the eight endpoints considered, and selected as the final predictive strategy for each type of toxicological prediction, we aimed at implementing an automated feature importance analysis able to highlight the structural moieties of a molecule that most contribute to a prediction of toxicity, thus providing information about the potential toxicophores of the molecule. The feature importance analysis was carried out according to the Shapley paradigm, which is a widely used approach to evaluate the impact of individual components on the final outcome derived from game theory.^10^ This methodology is applied to ML models with the purpose of evaluating the weight of each individual feature on the final prediction. In this work, the Shapley approach was applied to determine the magnitude of the impact of individual FP bits in the generation of the consensus predictions as summarized in Figure 5.

In particular, we developed a novel strategy for retro-mapping the results obtained by the Shapley method in order to highlight the structural moieties responsible for the predicted toxicity by employing an appropriate bit retro-mapping method for each FP present in the consensus combination. The atoms associated with individual FP bits were identified for each compound in accordance with the aforementioned protocols. A feature weighting method was then employed to assign a dependable score to each identified atom. In particular, the weight of the atom is given by the sum of the weights of the features in which it is contained. This weight is divided by the number of atoms in the feature and by the number of occurrences in the molecule. The final atom weight was computed as the average weight of each atom obtained from each model belonging to the best consensus approach. Finally, the mapping functions of RDKit were used to visualize the magnitude of the features that affect the toxicological prediction for a given molecule with respect to the requested endpoint (see Materials and Methods for details).

**Figure 6:**
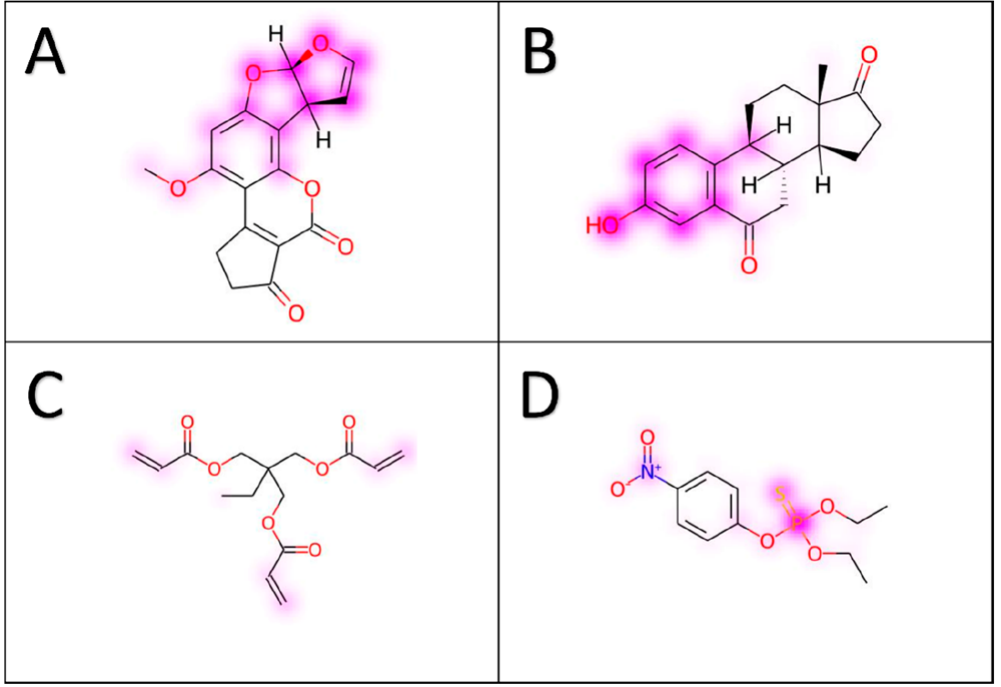
Results obtained from SHAP analysis for aflatoxin B1 (A), 6-ketoestrone (B), trimethylolpropane triacrylate (C), and ethyl-parathion (D).

In order to validate the reliability of our SHAP-based approach, we performed toxicity predictions and the corresponding feature importance analysis of compounds with known toxic effects and mechanism of action, which were not present in the training sets. Specifically, we tested the approach with four endpoints: carcinogenicity, estrogenicity, skin irritation, and acute oral toxicity. Concerning the carcinogenicity endpoint, we studied aflatoxin B1 (AFB1), a compound belonging to the aflatoxin family, produced by several strains of fungi, such as *Aspergillus flavus* and *A*. *parasiticus*, found as contaminants in a wide variety of crops, cereals, oilseeds, nuts and spices.^53,54^ The International Agency for Research on Cancer (IARC) classified AFB1 as a human carcinogen belonging to Group 1 because it contributes to liver cancer development by inducing the formation of DNA adducts. The consensus carcinogenicity prediction correcly predicted AFB1 as a carcinogen, and the corresponding features importance analysis obtained by the SHAP method suggested that the two five-membered heterocyclic rings, and especially the dihydrofuran moiety (Figure 6A), could be responsible for the carcinogenic activity. These results are in agreement with the literature and experimental data. Indeed, AFB1 is primarily metabolized in the liver by oxidase enzymes belonging to the CYP450 superfamily, by producing the reactive 8,9-epoxide, existing as two stereoisomers, exo and endo, with the former reported to be the toxic one. This epoxide form has a high binding affinity toward the DNA, forming the AFB1-N7-guanine DNA adduct, thus leading to DNA mutations.^55,56^

For the estrogenicity endpoint, we considered 6-ketoestrone, a potent inhibitor of 17β-hydroxysteroid dehydrogenase type 1 (17β-HSD),^57^ which is one of the steroidogenic enzymes that regulate the bioavailability of estrogens. One of the common structural features identified as responsible for ligand binding to the active site of 17β-HSD is the phenol moiety, which is involved in a hydrogen bond network with residues H221 and G282 of the protein.^58,59^Figure 6B shows that the consensus prediction identified 6-ketoestrone as an estrogenic compound and the SHAP analysis suggested the phenolic fragment as the moiety responsible for the toxicity of the molecule, in agreement with literature studies. We validated the SHAP method on the skin irritation endpoint studying trimethylolpropane triacrylate, which belongs to the family of acrylate derivatives. Such small chemicals can polymerize either spontaneously or with catalysts, such as ultraviolet (UV) light, giving rise to very resistant polymers. Acrylate monomers are potent sensitizing chemicals and cause allergic contact dermatitis (ACD).^60^ Also in this case, the compound was predicted to be a skin irritating agent, and the developed SHAP method evidenced the three acrilate groups as potential toxicophores, albeit with a slight intensity (Figure 6C). The last endpoint considered to verify the reliability of the SHAP method was the acute oral toxicity, for which ethyl-parathion was used as a test structure. This compound is a known organophosphate insecticide possessing an organothiophosphate group that generally disrupts nervous system cells by inhibiting acetylcholinesterase enzyme. Indeed, after ingestion of parathion, an oxidase enzyme replaces the double bonded sulfur with oxygen, thus producing paraoxon, which is more reactive in organisms than the phosphorothiolate ester. Such derivative acts as an acetylcholinesterase inhibitor, causing typical synptoms such as nausea and vomit, abdominal pain, diarrhea, and salivation.^61^ VenomPred 2.0 correctly predicted its acute oral toxicity and, based on the SHAP analysis, the sulfur–phosphorus bond was identified as the main contributor for the toxicity prediction of the compound (Figure 6D) in agreement with what reported in literature about the mechanism of action of organothiophospate compounds.

### Compared Performance Evaluations with Other Web Servers for Toxicological Predictions

With the aim of comparing the performance of VenomPred 2.0 with that of other machine learning web tools for *in silico* toxicity evaluations, we performed a literature search for identifying currently available Web servers that can be freely employed for toxicological predictions. Some of the identified tools could only perform predictions for a single molecule per query, thus making a robust statistical evaluation based on many compounds impractical. For this reason, we concentrated our attention on tools allowing users to perform batch predictions on multiple molecules for each query, i.e. ADMETlab 2.0,^62^ toxCSM,^63^ SSL-ToxGCN^64^ and ADMETSAR 2.0.^65^ Since ADMETlab 2.0 included all eight endpoints available in VenomPred 2.0, the test set compounds of each endpoint already employed for the evaluation of our platform were used to generate predictions with ADMETlab 2.0 and to evaluate the performance in terms of MCC. The obtained MCC values were then compared to those obtained using VenomPred 2.0 consensus predictions. The same approach was used for evaluating the predictive performance of toxCSM, except for the oral toxicity endpoint. Since the toxCSM model for oral toxicity is a regressor, whereas VenomPred 2.0 only uses binary classifiers, the compared performance assessment for this endpoint was not possible. For the other two Web servers, the reliability of predictions related to only two endpoints was assessed, since SSL-ToxGCN only shared with VenomPred 2.0 the androgenicity and estrogenicity endpoints (being focused on nuclear receptor and stress response toxicity), while ADMETSAR 2.0 is able to perform predictions for a maximum of 20 molecules per query. Therefore, the performance assessment of ADMETSAR 2.0 was restricted to two endpoints, whose test set employed for VenomPred 2.0 included a relatively smaller number of compounds. The results of this analysis, reported in Table 3, demonstrate that VenomPred 2.0 generally outperforms the other tested models, with dramatic improvements in terms of MCC for estrogenicity and androgenicity endpoints. Only for the carcinogenicity and mutagenicity endpoints ADMETlab 2.0 respectively showed similar and moderately higher performances compared to VenomPred 2.0. However, since the training sets used for generating the models of this tool were not available, we could not check whether molecules included in the test sets of VenomPred 2.0 were also present in the training sets of ADMETlab 2.0 model and thus whether the predictions were biased in some extent. On the contrary, although also toxCSM performed significantly better than VenomPred 2.0 on mutagenicity predictions, we could check that at least 85% of molecules included in the VenomPred 2.0 mutagenicity test set were used in the toxCSM training set, which implies that this result is certainly biased and overestimated. Overall, VenomPred 2.0 was demonstrated to represent a valuable and highly reliable platform for toxicity predictions.

**Table 3:** Performance Assessment of VenomPred 2.0 and Other Web Tools for Toxicological Predictions, Expressed in Terms of MCC, Related to Different End Points

End point	VenomPred 2.0	ADMETlab 2.0	toxCSM	SSL-ToxGCN	ADMETSAR 2.0
Carcinogenicity	0.50	0.51	0.14	n.d.^a^	n.d.
Mutagenicity	0.74	0.80	0.96	n.d.	n.d.
Hepatotoxicity	0.43	0.25	0.35	n.d.	0.42
Eye Irritation	0.49	0.43	0.44	n.d.	n.d.
Skin Irritation	0.58	0.19^b^	0.13^b^	n.d.	n.d.
Androgenicity	0.95	0.39	0.27	0.44	n.d.
Estrogenicity	0.84	0.52	0.36	0.34	0.30
Oral Toxicity	0.58	0.45	n.d.	n.d.	n.d.

a Not determined.

b These MCC values were obtained using models predicting skin sensitization effects of chemicals.

## Conclusions

The rapid evaluation of potential toxicological properties of small molecules through *in silico* studies represents an attractive field for drug discovery and safety assessments, especially in lead optimization and ADMET studies. In this context, we recently developed VenomPred, a free of charge web tool for toxicological predictions. Herein we present the development of VenomPred 2.0, the new upgraded version of our tool that now represents a powerful web-based platform for multifaceted and human interpretable toxicity predictions. VenomPred 2.0 maintains free and user-friendly features, thus allowing its full accessibility even to inexperienced users, and presents a doubled set of toxicity endpoints that can be evaluated, namely, carcinogenicity, mutagenicity, hepatotoxicity, estrogenicity, androgenicity, skin irritation, eye irritation, and acute oral toxicity. Notably, an innovative exhaustive consensus strategy, based on the combined use of multiple ML models, was applied both for maximizing the performance of the toxicity predictions of the four newly added endpoints and for further improving the reliability of the already available endpoints, which makes VenomPred 2.0 a fully new *in silico* toxicological platform. Moreover, we also implemented a new utility based on the Shapley Additive exPlanations (SHAP) method that confers human interpretability to VenomPred 2.0 predictions, enabling the exploration of the specific structural moieties that are associated with the predicted toxicological effects of a molecule. This way, by simply loading on our platform (http://www.mmvsl.it/wp/venompred2/) the SMILES strings of the desired compounds, which can also be obtained by easily drawing the corresponding molecular structures on the platform sketcher, the user can easily and rapidly obtain information about the toxicological potential of the desired molecules and about the possible toxicophores and structural fragments responsible for their toxicity (Figure S3). Finally, it is worth mentioning that the models integrated in our original VenomPred platform are also implemented in our software MolBook UNIPI, which is a powerful, free-of-charge and easy-to-use tool for managing virtual libraries of chemical compounds.^66^ MolBook UNIPI users can predict the potential carcinogenic, mutagenic, hepatotoxic, and estrogenic effect of their compound libraries locally, by using the VenomPred models integrated in the software, in a very simple and fast way that requires neither programming skills and chemoinformatics knowledge nor an Internet connection. Future versions of MolBook UNIPI will include the new models integrated in the VenomPred 2.0 platform.

## Data Availability

Information about the data sets used for developing androgenicity, skin irritation, eye irritation and acute oral toxicity models, as well as about the hyperparameters settings evaluated during their optimization process, is reported within the Materials and Methods section of the manuscript. The MCC values achieved in the test set prediction by all optimized androgenicity, skin irritation, eye irritation and acute oral toxicity models, are reported within the Supporting Information (Figure S2). Information about the data sets used for the previously developed mutagenicity, carcinogenicity, estrogenicity and hepatotoxicity models, as well as about the performance of the best model for each of these four toxicity endpoints, is reported within the Supporting Information (Tables S1–2). Information about the models included in the best consensus combination herein identified for each of the eight total toxicity endpoints considered, as well as about the predictive performance of the corresponding consensus predictions, are reported within the Supporting Information (Table S3). Information about the determination of features contribution, the bit retro-mapping method for analyzing feature importance is reported within the Materials and Methods section of the manuscript. VenomPred 2.0 platform is accessible as a free of charge web tool at the following page: http://www.mmvsl.it/wp/venompred2/. The trained models integrated in VenomPred 2.0, together with the training and test sets respectively used to develop and evaluate them, as well as the related APIs, are also freely available at the following GitHub page: https://github.com/MMVSL/VenomPred2.0.

## Supporting Information Available

Table S1:Training and test set composition employed for generating models for mutagenicity, carcinogenicity, estrogenicity and hepatotoxicity endpoints; Figure S1: Overlaid projections of the training and test set compounds related to each endpoint in the same two-dimensional space; Figure S2: MCC results obtained for the whole set of models generated for androgenicity, skin irritation, eye irritation and acute oral toxicity endpoints; Table S2: Performance results obtained for the top-score models of mutagenicity, carcinogenicity, estrogenicity and hepatotoxicity endpoints; Table S3: Performance results achieved by the best consensus combination for each endpoints; Figure S3: VenomPred 2.0 panel for loading and evaluating compounds; Scikit-learn: a python library for machine learning; Grid-search and hyperparameters optimization; Permutation test (y-randomization); Applicability domain; References (PDF)
