# Supplementary material for: VenomPred 2.0: A Novel In Silico Platform for an Extended and Human Interpretable Toxicological Profiling of Small Molecules
Source: J Chem Inf Model. 2023 Sep 7;64(7):2275–89. doi: 10.1021/acs.jcim.3c00692 (PMC11005041; doi:10.1021/acs.jcim.3c00692)
Supplement: Supplementary file 1 — ci3c00692_si_001.pdf [file ci3c00692_si_001.pdf]

# Supporting Information

# VenomPred 2.0: a Novel *In Silico* Platform for an Extended and Human Interpretable Toxicological Profiling of Small Molecules

*Miriana Di Stefano[a,b], Salvatore Galati[a], Lisa Piazza[a], Carlotta Granchi[a], Simone Mancini[c], Filippo Fratini[c], Marco Macchia[a], Giulio Poli[a,*], Tiziano Tuccinardi[a]*

[a] Department of Pharmacy, University of Pisa, Via Bonanno 6, 56126 Pisa, Italy. [b] Department of Life Sciences, University of Siena, 53100 Siena, Italy. [c] Department of Veterinary Sciences, University of Pisa, Viale Delle Piagge 2, 56124, Pisa, Italy.

Address correspondence to: Giulio Poli, giulio.poli@unipi.it

### Table of Contents

**Table S1.** Training and test set composition employed for generating models for mutagenicity, carcinogenicity, estrogenicity and hepatotoxicity endpoints. **S3**

**Figure S1**. Overlaid projections of the training and test set compounds related to each endpoint in the same two-dimensional space **S4**

**Figure S2.** MCC results obtained for the whole set of models generated for androgenicity, skin irritation, eye irritation and acute oral toxicity endpoints **S5**

**Table S2.** Performance results obtained for the top-score models of mutagenicity, carcinogenicity, estrogenicity and hepatotoxicity endpoints **S6**

**Table S3.** Performance results achieved by the best consensus combination for each endpoint **S6**

**Figure S3.** VenomPred 2.0 panel for loading and evaluating compounds **S7**

**Scikit-learn: a python library for machine learning S8**

**Grid-search and hyperparameters optimization S9**

**Permutation test (y-randomization) S10**

**Applicability domain S11**

**References S12**

**Table S1**. Total number of molecules present in training and test sets employed for generating mutagenicity, carcinogenicity, estrogenicity and hepatotoxicity models.

| **Mutagenicity Model** | | | |
|---|---|---|---|
| Data Set | Total | Non-mutagen | Mutagen |
| Training | 3367 | 1484 | 1883 |
| Test | 798 | 352 | 446 |
| **Carcinogenicity Model** | | | |
| Data Set | Total | Non-Carcinogenic | Carcinogenic |
| Training | 645 | 312 | 333 |
| Test | 161 | 72 | 89 |
| **Estrogenicity Model** | | | |
| Data Set | Total | Inactive | Active |
| Training | 656 | 422 | 234 |
| Test | 150 | 96 | 54 |
| **Hepatotoxicity Model** | | | |
| Data Set | Total | Non-Toxic | Toxic |
| Training | 760 | 352 | 408 |
| Test | 157 | 60 | 97 |

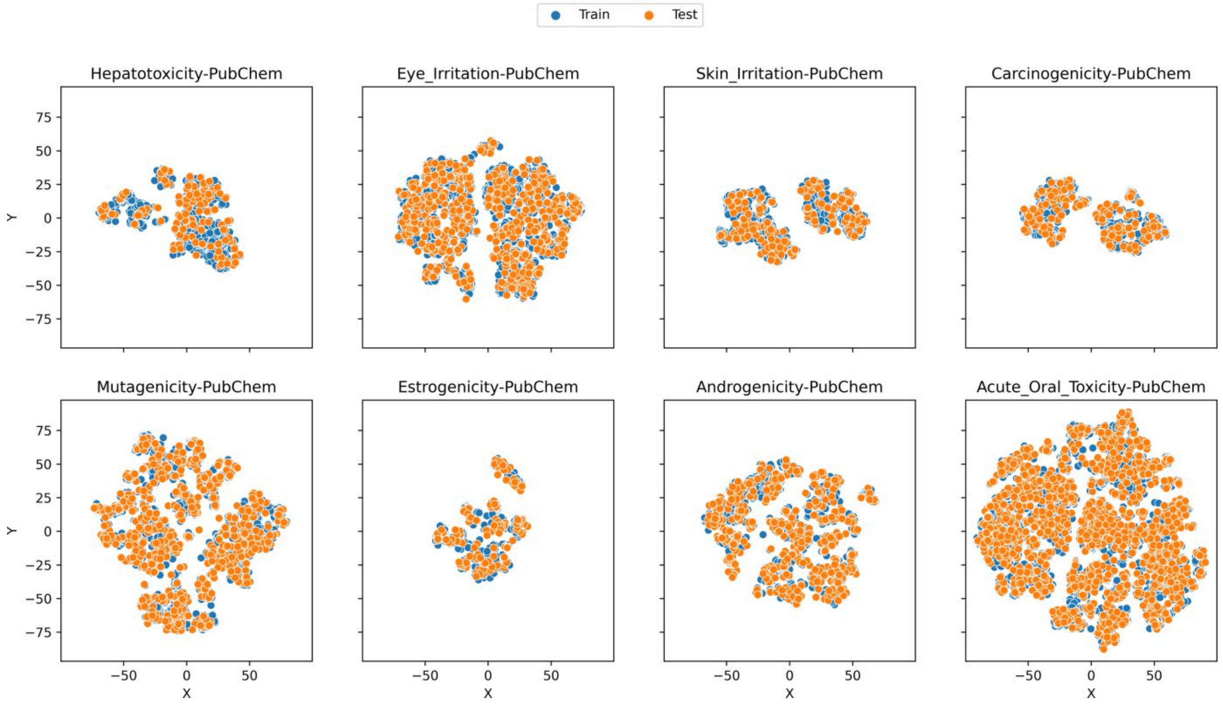


**Figure S1.** Overlaid projections of the training and test set compounds related to each endpoint in the same two-dimensional space. The dimensionality reduction was obtained applying the t-distributed stochastic neighbor embedding (t-SNE) algorithm on the compounds encoded as PubChem FPs.

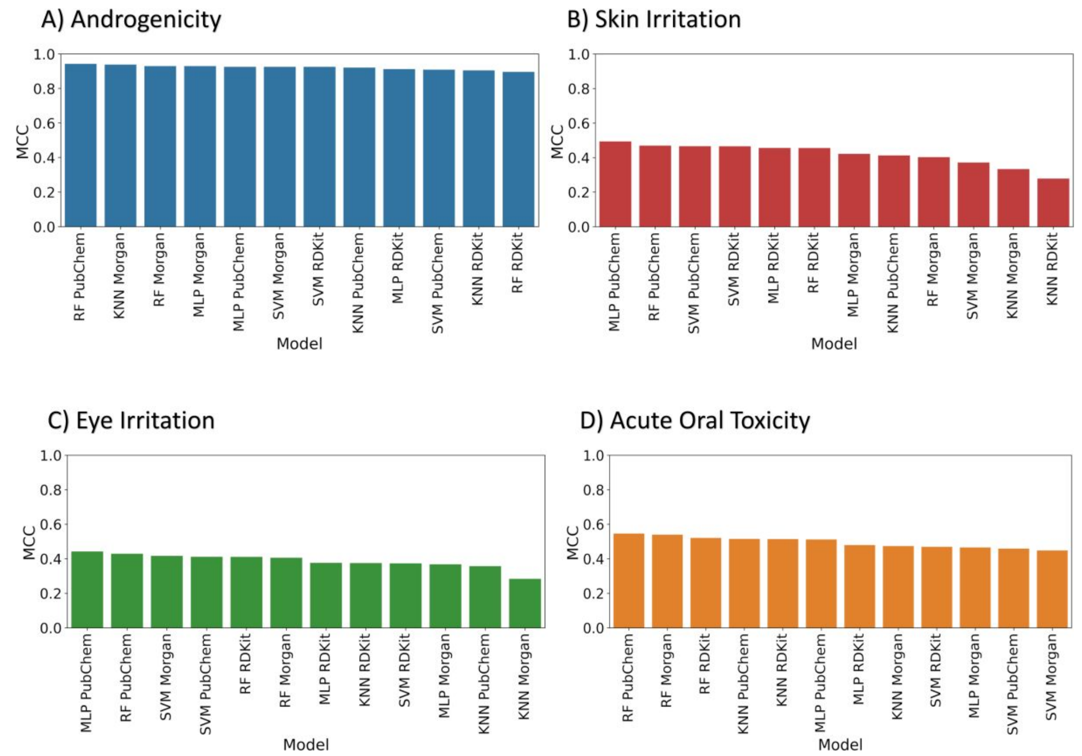


**Figure S2.** Performance evaluation results, based on test set prediction, in terms of MCC obtained for the whole set of models generated for A) androgenicity, B) skin irritation, C) eye irritation and D) acute oral toxicity endpoints.

**Table S2**. Models performance measured as MCC, Recall, Precision, Specificity and Accuracy for Mutagenicity, Carcinogenicity, Estrogenicity and Hepatotoxicity endpoints obtained with the original top scored models from VenomPred.

| Endpoints | Model | MCC | Recall | Precision | Specificity | Accuracy |
|---|---|---|---|---|---|---|
| Mutagenicity | RF- Pubchem | 0.69 | 0.85 | 0.87 | 0.84 | 0.84 |
| Carcinogenicity | RF- Pubchem | 0.39 | 0.72 | 0.73 | 0.67 | 0.70 |
| Estrogenicity | RF- Pubchem | 0.83 | 0.85 | 0.92 | 0.96 | 0.92 |
| Hepatotoxicity | SVM-Morgan | 0.26 | 0.86 | 0.69 | 0.37 | 0.67 |

**Table S3**. Models performance measured as MCC, Recall, Precision, Specificity and Accuracy for the best new consensus strategy of all considered toxicological endpoints.

| Endpoints | Model Combinations | MCC | Recall | Precision | Specificity | Accuracy |
|---|---|---|---|---|---|---|
| Androgenicity | MLP_PubChem, SVM_PubChem, KNN_Morgan, RF_RDKit, RF_Morgan, SVM_RDKit | 0.95 | 0.93 | 1.00 | 1.00 | 0.98 |
| Eye irritation | MLP_PubChem, RF_Morgan ,KNN_Morgan, RF_PubChem | 0.49 | 0.68 | 0.64 | 0.80 | 0.77 |
| Skin irritation | SVM_PubChem, RF_Morgan, MLP_RDKit, RF_PubChem | 0.58 | 0.67 | 0.73 | 0.89 | 0.83 |
| Acute Oral toxicity | SVM_PubChem, KNN_RDKit, RF_Morgan, MLP_Morgan, KNN_Morgan, KNN_PubChem, RF_PubChem ,RF_RDKit, MLP_PubChem | 0.58 | 0.84 | 0.80 | 0.74 | 0.8 |
| Mutagenicity | KNN_RDKit, RF_PubChem, SVM_PubChem, RF_Morgan, SVM_RDKit, SVM_Morgan | 0.74 | 0.89 | 0.88 | 0.85 | 0.86 |
| Carcinogenicity | KNN_Morgan, KNN_PubChem, RF_RDKit, RF_Morgan, SVM_PubChem, KNN_RDKit, SVM_RDKit | 0.50 | 0.74 | 0.80 | 0.76 | 0.75 |
| Estrogenicity | SVM_PubChem, RF_PubChem, SVM_RDKit | 0.84 | 0.91 | 0.90 | 0.93 | 0.92 |
| Hepatotoxicity | SVM_PubChem, RF_RDKit, SVM_RDKit, MLP_Morgan | 0.43 | 0.97 | 0.71 | 0.35 | 0.73 |

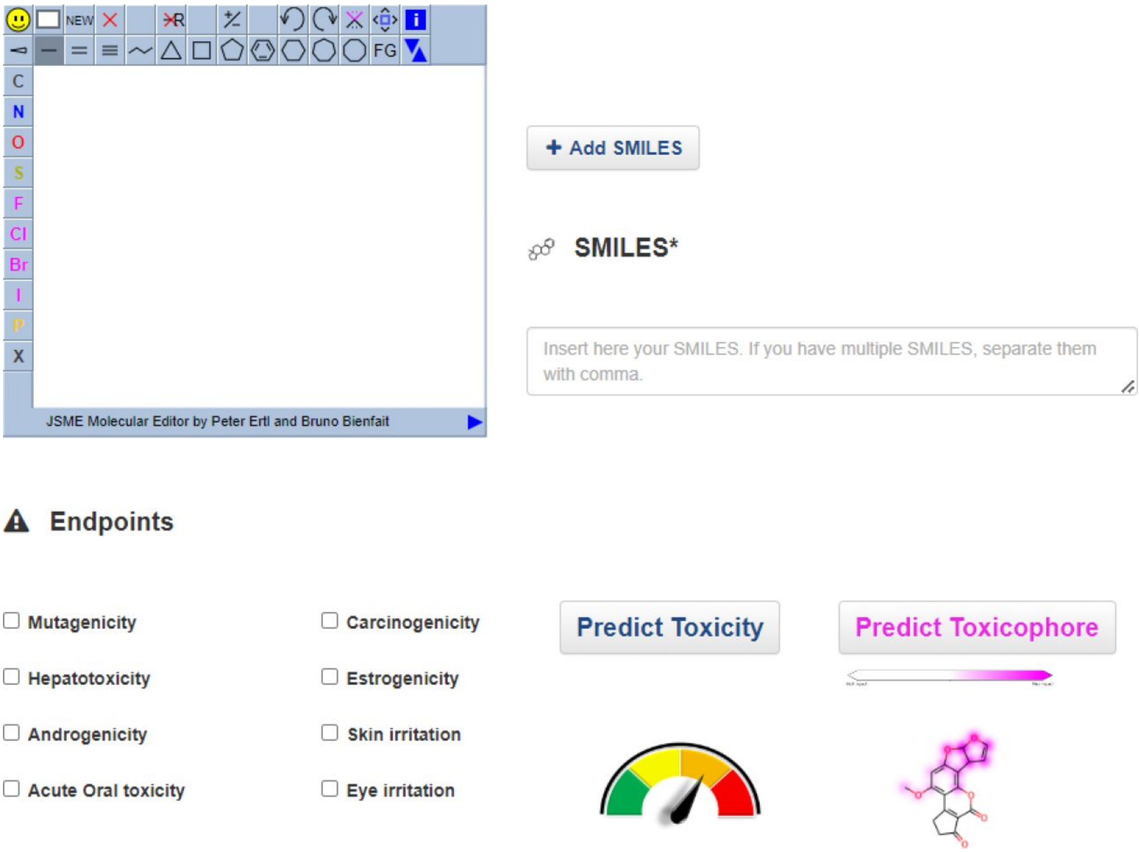


**Figure S3.** VenomPred 2.0 panel for loading compounds and selecting the desired toxicity evaluations.

**Scikit-learn: a python library for machine learning**

Scikit-learn is a popular open-source machine learning library for Python.[1] It provides a wide range of tools and algorithms for tasks such as classification, regression, clustering, dimensionality reduction, and model selection. Scikit-learn is designed to be user-friendly and efficient, offering a robust API and extensive documentation. It integrates well with other popular Python libraries like NumPy, SciPy, and Pandas, making it a powerful tool for data analysis and machine learning. Scikit-learn offers a rich collection of machine learning algorithms, provides a variety of evaluation metrics and techniques for assessing the performance of machine learning models, and includes functions for cross-validation, hyperparameter tuning and model selection.

## Grid search and hyperparameters optimization

Grid search is a widely used hyperparameter optimization technique in machine learning and it involves creating a "grid" of hyperparameter combinations and exhaustively exploring them to find the optimal set of hyperparameters that yields the best performance for a given model and dataset.[2] The approach is important since the hyperparameters could significantly influence the performance of the models. After the grid search step, we obtained optimized models with higher MCC values (see Table 2 in the main text) than those shown by the initial models with non-optimized hyperparameters (reported below, in Table S4) for the prediction of the test set compounds. Nevertheless, no dramatic change in terms of performance was observed between non-optimized and optimized models, as expected due to the general suitability of the sets of hyperparameters employed as a starting point of each model.

**Table S4.** Performance evaluation results, based on test set prediction, obtained for the top-scored (non-optimized) models in terms of MCC for androgenicity, eye irritation, skin irritation and acute oral toxicity endpoints.

| *Endpoint* | *Model* | *MCC* | *Precision* | *Recall* | *Specificity* | *Accuracy* |
|---|---|---|---|---|---|---|
| Androgenicity | RF_PubChem | 0.93 | 0.97 | 0.93 | 0.98 | 0.97 |
| Eye Irritation | MLP_PubChem | 0.40 | 0.59 | 0.60 | 0.80 | 0.73 |
| Skin Irritation | MLP_PubChem | 0.48 | 0.63 | 0.65 | 0.83 | 0.77 |
| *Acute* Oral Toxicity | RF_PubChem | 0.54 | 0.78 | 0.83 | 0.70 | 0.77 |

**Permutation test (y-randomization)**

The permutation test, also known as the randomization test or exact test, offers a non-parametric approach to hypothesis testing. Unlike conventional parametric methods that assume specific data distributions, the permutation test sidesteps such assumptions, making it well-suited for assessing the significance of models applied to diverse and complex datasets. Its robustness against data irregularities and its ability to handle small sample sizes render it a preferred choice in scenarios where traditional statistical tests may be limited or yield misleading results. We performed the permutation test for each of the 12 models obtained for each endpoint considered in the exhaustive consensus analysis, in order to assess the ability of the models to correctly detect patterns of information that can correlate the chemical features to the toxicological profiles of the compounds. The results of this analysis, reported below in Table S5, strongly confirmed the reliability of our models. In fact, as expected, MCC values of zero and Accuracy values around 0.50, indicating completely random predictions, were obtained as a result of the randomization tests. The analysis highlights that the performances of our trained models are not due to chance, since in the absence of correlation between toxicity and chemical-structural information of the compounds (as obtained through y-randomization), the prediction of the models is totally random.

**Table S5**: Results of permutation test for each endpoint in terms of MCC and Accuracy. For both statistical metrics, the related standard deviation is also reported.

| *Endpoint* | *MCC* | *Accuracy* |
|---|---|---|
| Hepatotoxicity | 0.00 +/- 0.01 | 0.51 +/- 0.01 |
| Eye Irritation | 0.00 +/- 0.01 | 0.54 +/- 0.01 |
| Skin Irritation | 0.00 +/- 0.01 | 0.55 +/- 0.01 |
| Carcinogenicity | 0.00 +/- 0.01 | 0.54 +/- 0.01 |
| Mutagenicity | 0.00 +/- 0.01 | 0.54 +/- 0.01 |
| Estrogenicity | 0.00 +/- 0.01 | 0.54 +/- 0.01 |
| Androgenicity | 0.00 +/- 0.01 | 0.55 +/- 0.01 |
| Acute Oral Toxicity | 0.00 +/- 0.01 | 0.55 +/- 0.01 |

## Applicability domain

In the context of machine learning, the definition of a confidence space is important to provide the confidence associated to the models' predictions. Such space, commonly named Applicability Domain (AD), represents an appropriate measure of goodness-of-fit to the predictions of new instances. Predictions made by interpolations within the model's AD, as occurs for compounds with high structural similarity to the training data, are considered highly reliable. On the other hand, predictions made by extrapolation beyond the model's AD are expected to be less reliable. In this work, we used a distance-based approach involving tanimoto similarity to define a possible AD of our models. This method consists in an initial search for the 5 compounds of the training set that are most similar to an external predicted compound. The average similarity between the predicted instance and the 5 most similar training compounds (dTC) is then compared with the average similarity between the training compound and its 5 nearest neighbors (dNN).[3] A predicted instance is considered within the AD if the dTC is greater than or equal to the dNN. In this case, the new molecule is closely related to the compounds in the training set. On the other hand, if the dTC is lower than dNN, the molecules are less structurally similar to the training compounds and thus the prediction may not have a high confidence. To verify the applied approach, we evaluated the performance of the models on two different groups of test set compounds, identified based on the defined AD. Specifically, each molecule in the test set was labelled as 'In' if it fell within the above defined AD or 'Out' if it remained outside the AD (Figure S4**).** The results show that, as expected, considering only the 'In' predictions, the performance of the models is generally better than the 'Out' predictions. Nevertheless, except for the hepatotoxicity endpoint, no dramatic decrease in terms of MCC is observed.

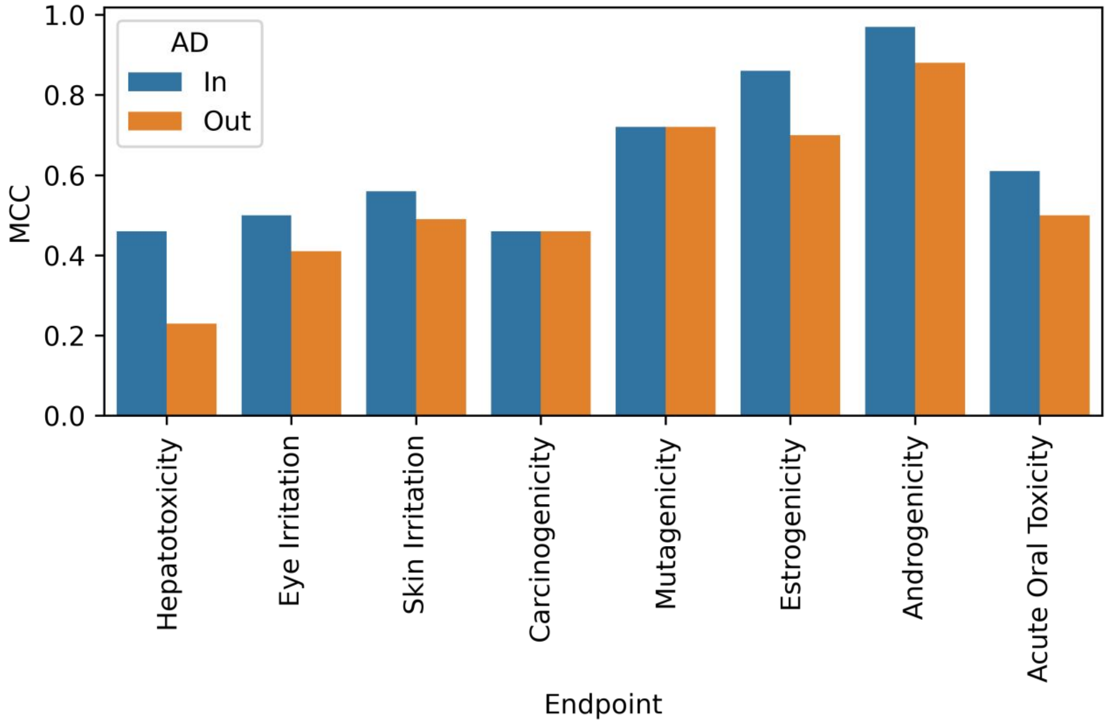


**Figure S4**: Predictive performance expressed in terms of MCC, obtained for compounds located within (In) and outside (out) the AD using the best consensus prediction for each endpoint.

## References

(1) Pedregosa, F.; Varoquaux, G.; Gramfort, A.; Michel, V.; Thirion, B.; Grisel, O.; Blondel, M.; Prettenhofer, P.; Weiss, R.; Dubourg, V.; et al. Scikit-Learn: Machine Learning in Python. *J. Mach. Learn. Res.* **2011**, *12*, 2825–2830.

(2) Hou, Q.; Waury, K.; Gogishvili, D.; Feenstra, K. A. Ten Quick Tips for Sequence-Based Prediction of Protein Properties Using Machine Learning. *PLOS Comput. Biol.* **2022**, *18* (12), e1010669.

(3) Kar, S.; Roy, K.; Leszczynski, J. Applicability Domain: A Step Toward Confident Predictions and Decidability for QSAR Modeling. In *Methods in Molecular Biology*; 2018; pp 141–169.
